# Hospitalization of patients with nutritional anemia in the United States in 2020

**DOI:** 10.3389/fpubh.2024.1333069

**Published:** 2024-05-13

**Authors:** Jie Tian, YangYang Fan, Xin Wei, Jiangli Li, ZeLong Yang, Xiaolin Na, Yunbo Zhang

**Affiliations:** Department of Environmental Hygiene, Public Health College, Harbin Medical University, Harbin, Heilongjiang, China

**Keywords:** HCUP NIS, nutritional anemia, hospitalization, public health, iron deficiency anemia

## Abstract

**Background:**

Nutritional anemia is highly prevalent and has triggered a globally recognized public health concern worldwide.

**Objective:**

To better understand the prevalence of anemia and the state of nutritional health in developed countries to inform global nutritional health and better manage the disease.

**Method:**

We employed the Healthcare Cost and Utilization Project (HCUP)-2020 National Inpatient Health Care Data (NIS), administered by The Agency for Healthcare Research and Quality. Nutritional anemia was diagnosed according to the International Classification of Diseases, 10th Revision (ICD-10). Matching analysis and multivariate regression were used to adjust for patient and hospital characteristics. Controls were obtained by stratifying and matching for age and sex.

**Results:**

The 2020 HCUP-NIS database encompassed a survey over 6.4 million hospitalized patients, among which 1,745,350 patients diagnosed with anemia, representing approximately 26.97% of the hospitalized population, over 310,000 were diagnosed with nutritional anemia, and 13,150 patients were hospitalized for nutritional anemia as primary diagnosis. Hospitalization rate for nutritional anemia exhibited an increased age-dependent increase nationwide, especially among females, who displayed 1.87 times higher than males. Notably, in comparison to the control group, individuals of the Black race exhibit a higher prevalence of nutritional anemia (case group: 21.7%, control group: 13.0%, *p* < 0.001). In addition, hospitalization rates were higher among low-income populations, with lower rates of private insurance (case group: 18.7%, control group: 23.5%, *p* < 0.001) and higher rates of Medicaid insurance (case group: 15.4%, control group: 13.9%, *p* < 0.001). In areas characterized by larger urban centers and advanced economic conditions within the urban–rural distribution, there was an observed increase in the frequency of patient hospitalizations. Iron deficiency anemia emerged as the predominant subtype of nutritional anemia, accounting for 12,214 (92.88%). Secondary diagnosis among patients hospitalized for nutritional anemia revealed that a significant number faced concurrent major conditions like hypertension and renal failure.

**Conclusion:**

In economically prosperous areas, greater attention should be given to the health of low-income individuals and the older adult. Our findings hold valuable insights for shaping targeted public health policies to effectively address the prevalence and consequences of nutritional anemia based on a overall population health.

## 1 Introduction

Nutritional anemia is a disease characterized by inadequate formation of hemoglobin or the production of red blood cells is insufficient due to the relative or absolute reduction in essential nutrients required for blood production within the body, such as iron, folic acid, and vitamin D, resulting in low hematopoietic function (1). This condition is most prevalent among infants and young children aged 6 months to 2 years (2), pregnant or lactating women, and individuals with impaired nutrients absorption due to gastrointestinal disorders (3–5). Nutritional anemia can lead to a lack of oxygen in the body, causing the patient to feel tired, weak, and experience symptoms such as dizziness, panic, and shortness of breath. Severe anemia may affect the functioning of the heart, lungs, and other organs, leading to serious problems such as palpitations, angina, and difficulty breathing (6, 7). Nutritional anemia during childhood may lead to poor concentration, reduced learning ability, and delayed mental development (8). Nutritional anemia in pregnant women increases the risk of preterm birth, low birth weight and fetal growth retardation and affects the normal functioning of the immune system, reducing the body’s resistance to disease and increasing the risk of infection (9, 10).

The World Health Organization estimates that approximately a quarter of the global population is suffering from anemia (11). Nutritional anemia tends to be underestimated due to absence of immediate life-threatening consequences, leading to reduced attention from both the individuals and the general public. In 2021, anemia affected approximately 24.3% of the global population across all age groups with totaling 1.92 billion cases, contrasting with rates of 28.2% and 1.50 billion cases recorded in 1990 (12). Although the United States has a lower incidence of anemia compared to the global average, a previous research based on the National Health and Nutrition Examination Survey (NHANES) database indicated an almost doubling of cases, rising from 4.03% in 1999 to 6.49% in 2020 (13). Other studies (14, 15) have also shown that the prevalence of anemia in the U.S. has been increasing every year for the last 20 years, which serves as a warning highlighting the need to pay greater attention to anemia. However, its sample size was relatively small, and it lacked detailed information including subtypes of anemia, the causes of anemia as well as the hospitalization and care of patients. In order to better understand the epidemiological characteristics of nutritional anemia as well as medical treatment in the United States, we performed a comprehensive analysis of nutritional anemia based on the Healthcare Cost and Utilization Project (HCUP)-2020 National Inpatient Medical Data (NIS), which is managed by the Agency for Healthcare Research and Quality (AHRQ). The aim of present study is to offer valuable recommendations to governments and the general public, contributing to the enhancement of human health.

## 2 Methods

### 2.1 Data source

For research purposes, we analyzed the 2020 HCUP-NIS database. The HCUP is a database series developed under the auspices of the Agency for Healthcare Research and Quality (AHRQ) that contains all patient encounter, clinical, and nonclinical information since 1988. It is the largest publicly available database of inpatient care information available in the United States. The HCUP-NIS contains discharge data from more than 1,000 hospitals and represents a stratified sample of 20% of community hospitals, which enable research on a wide range of health policy issues, including cost and quality of health services, medical practice patterns, access to health care programs, treatment outcomes at the national, state, and local levels.

### 2.2 Study design and sample

This study conducted a descriptive analysis of all patients hospitalized for nutritional anemia in the 2020 HCUP-NIS dataset. The dataset contained a total of 6,471,165 hospitalized patients, in which nutritional anemia was diagnosed according to the International Classification of Diseases, Tenth Revision (diagnostic codes: ICD-10/D50~D53, See Supplementary materials for details), and a total of 13,150 patients with the first diagnosis (hospitalized for nutritional anemia) were included. Since age and gender are extremely important influences on nutritional anemia, adjustments were made to mitigate their effects on other variables. Due to the absence of gender information for one case in 13,150 patients, we could only match 13,149 patients in a ratio of 1:5 stratified by age and sex (16), resulting in a total of 65,745 controls (individuals not suffering from nutritional anemia) being included in present study. All patient data have been de-identified.

### 2.3 Element descriptions

We did a systematic analysis of variables such as age, gender, race, income, insurance class, patient care, and location of the patient for control and case groups included in the analysis. Age is classified according to the age segment of the HCUP database itself: < 1 year, 1–17 years, 18–44 years, 45–64 years and ≥ 65 years. Gender was classified into male and female. Racial classification included White, Black, Hispanic, Asian/Pacific Islander, Native American, and Other. Median household income national quartile annual variance for patient was classified as First quartile (1–49,999), Second quartile (50000–64,999), Third quartile (65000–85,999), and Fourth quartile (>86,000). Type of insurance was classified as Medicare, Medicaid, Private insurance, Self-pay, No charge, and Other. Location of patient was classified as Large Central Metro (≥ 1 million population), Large Fringe Metro (≥ 1 million population), Medium Metro (250000–999,999 population), Small Metro (50000–249,999 population), Micropolitan, and Noncore. Patient care status was classified as Discharged to home or self care, Transfer: short-term hospital, Transfer: other type of facility, Home health care, against medical advice, Died in hospital, Discharged alive, and destination unknown. Location of Hospital was classified as New England, Middle Atlantic, East North Central, West North Central, South Atlantic, East South Central, West South Central, Mountain, and Pacific.

### 2.4 Statistical analysis

The data were processed using the official website SAS software code after obtaining the official license from HCUP. All data were statistically analyzed by SPSS version 26. Means (M) and standard deviations (SD) were calculated and T-tests were used for continuous variables, and frequencies and percentages were presented for categorical variables. Differences between the control and case groups were assessed by chi-square test (*p value* < 0.05 as statistically significant). Regression analysis was used to adjust for each hospital variable. Tool GraphPad Prism 8 for graphic design.

## 3 Results

### 3.1 Nutritional anemia prevalence trends 2016–2020

In 2020, there were a total of 6,471,165 hospitalizations in the United States HCUP database. About 1,745,350 patients diagnosed with anemia, representing approximately 26.97% of the hospitalized population. Among them, a total of 315,004 were diagnosed with nutritional anemia (N for All-Listed). The prevalence was 4.87%, implying that 4–5 out of every 100 hospitalizations were for nutritional anemia. In addition, 13,150 were hospitalized for nutritional anemia as the first diagnosis (N for DX1).

**Figure 1:**
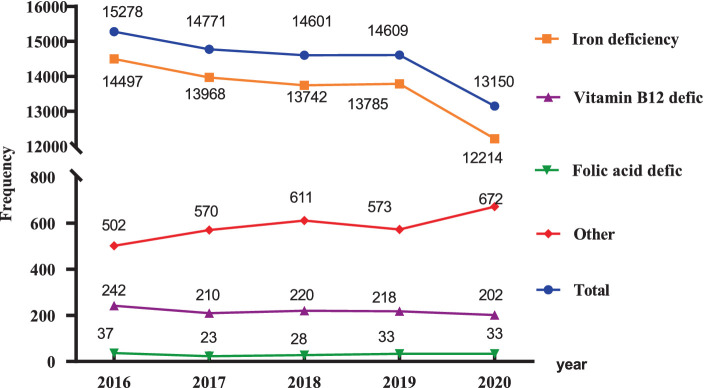
Nutritional anemia trend graph from 2016 to 2020.

Since 2016, the number of hospitalizations for patients with nutritional anemia has slightly trended downward, from 15,278 in 2016 to 13,150 in 2020, a decrease of about 13.93% (Figure 1). Among all the types of nutritional anemia, iron deficiency anemia was the main type of anemia accounting for 92.88%, while other types nutritional anemia such as folic acid, Vitamin B12, and other nutrient deficiencies resulting in anemia accounted for only 7.12% in 2020.

### 3.2 Demographic and statistical characteristics

The average age of patients hospitalized with nutritional anemia as the first diagnosis was 63.34 years (95% confidence interval: 62.99–63.66), and the mean length of hospitalization was 3.29 days (95% CI: 3.23–3.34), average cost per hospitalized patient was $39,147.07. Both the number of hospitalized patients and the length of hospitalization increased with advancing age. Among all the patients with nutritional anemia, the number of female patients was 1.88 times higher than male patients, and over half of the hospitalized patients were older adult, with 56.5% being older than 65 years (Table 1). There were relatively more Black and Hispanic individuals in the case group compared to controls, with the primary contrast observed between Black and White individuals (White: 60.4% vs. 71.1%; Black: 21.7% vs. 13.0%, *p* < 0.001). Income and nutritional anemia prevalence were negatively correlated, with higher prevalence in the low-income group. In addition, among payment types, the case group had a relatively high proportion of Medicare and Medicaid coverage and a relatively low proportion of private coverage (18.7% vs. 23.5%, *p* < 0.001). Compared to the control group, patients with nutritional anemia showed a higher inclination towards opting self-care at home after discharge from the hospital (70.1% vs. 60.1, *p* < 0.001) rather than continuing treatment in other health care units or choosing home care health care. The data showed that larger cities tended to have a higher incidence of nutritional anemia, with large central cities accounting for 31.2% of the cases, compared to only 6.7% in non-core cities.

**Table 1:** Study sample characteristics of nutritional anemia (case group vs. matched group).

Characteristic	Individuals with a diagnosis of nutritional anemia	Effective percentage	Individuals without a diagnosis of nutritional anemia	Effective percentage	*p*
Age in years, *n* (%)					1.00
<1	18	0.2	90	0.2	
1–17	474	3.6	2,370	3.6	
18–44	1884	14.3	9,420	14.3	
45–64	3,345	25.4	16,725	25.4	
≥65	7,429	56.5	37,140	56.5	
Gender, *n* (%)					1.00
Female	8,581	65.3	42,905	65.3	
Race, *n* (%)					<0.001
White	7,766	60.4	45,547	71.1	
Black	2,787	21.7	8,303	13.0	
Hispanic	1,508	11.7	6,425	10.0	
Asian/Pacific Islander	303	2.4	1,588	2.5	
Native American	82	0.6	370	0.6	
Other	403	3.1	1860	2.9	
Annual median household income national quartile, *n* (%)					<0.001
1–49,999	4,419	34.1	19,087	29.5	
50,000–64,999	3,419	26.4	17,618	27.2	
65,000–85,999	2,822	21.8	14,994	23.2	
>86,000	2,283	17.6	12,988	20.1	
PAY, *n* (%)					<0.001
Medicare	7,585	57.7	36,948	56.3	
Medicaid	2022	15.4	9,136	13.9	
Private insurance	2,451	18.7	15,460	23.5	
Self-pay	728	5.5	2070	3.2	
No charge	66	0.5	165	0.3	
Other	288	2.2	1883	2.9	
Disposition of patient (uniform), *n* (%)					<0.001
Discharged to home or self care	9,209	70.1	39,497	60.1	
Transfer:short-term hospital	146	1.1	1,325	2.0	
Transfer: other type of facility	1,581	12.0	10,429	15.9	
Home health care	1804	13.7	11,460	17.4	
Against medical advice	340	2.6	954	1.5	
Died in hospital	66	0.5	2028	3.1	
Discharged alive, Destination unknown	–*	-	22	0.0	
Patient location, *n* (%)					<0.001
Large Central Metro	4,080	31.2	17,977	27.5	
Large Fringe Metro	3,272	25.0	15,913	24.3	
Medium Metro	2,727	20.9	13,920	21.3	
Small Metro	1,089	8.3	6,327	9.7	
Micropolitan	1,037	7.9	6,350	9.7	
Noncore	871	6.7	4,927	7.5	
Census division of hospital, *n* (%)					<0.001
New England	676	5.1	3,378	5.1	
Middle Atlantic	2038	15.5	9,075	13.8	
East North Central	1868	14.2	9,617	14.6	
West North Central	770	5.9	4,749	7.2	
South Atlantic	3,113	23.7	13,947	21.2	
East South Central	948	7.2	4,081	7.1	
West South Central	1,683	12.8	7,944	12.1	
Mountain	525	4.0	4,266	6.5	
Pacific	1,529	11.6	8,088	12.3	
Hospitalizations, *n* (%)					
Total	13,150	100	65,745	100	

^*^indicates a value less than 10.

### 3.3 Distribution of patients by region

**Figure 2:**
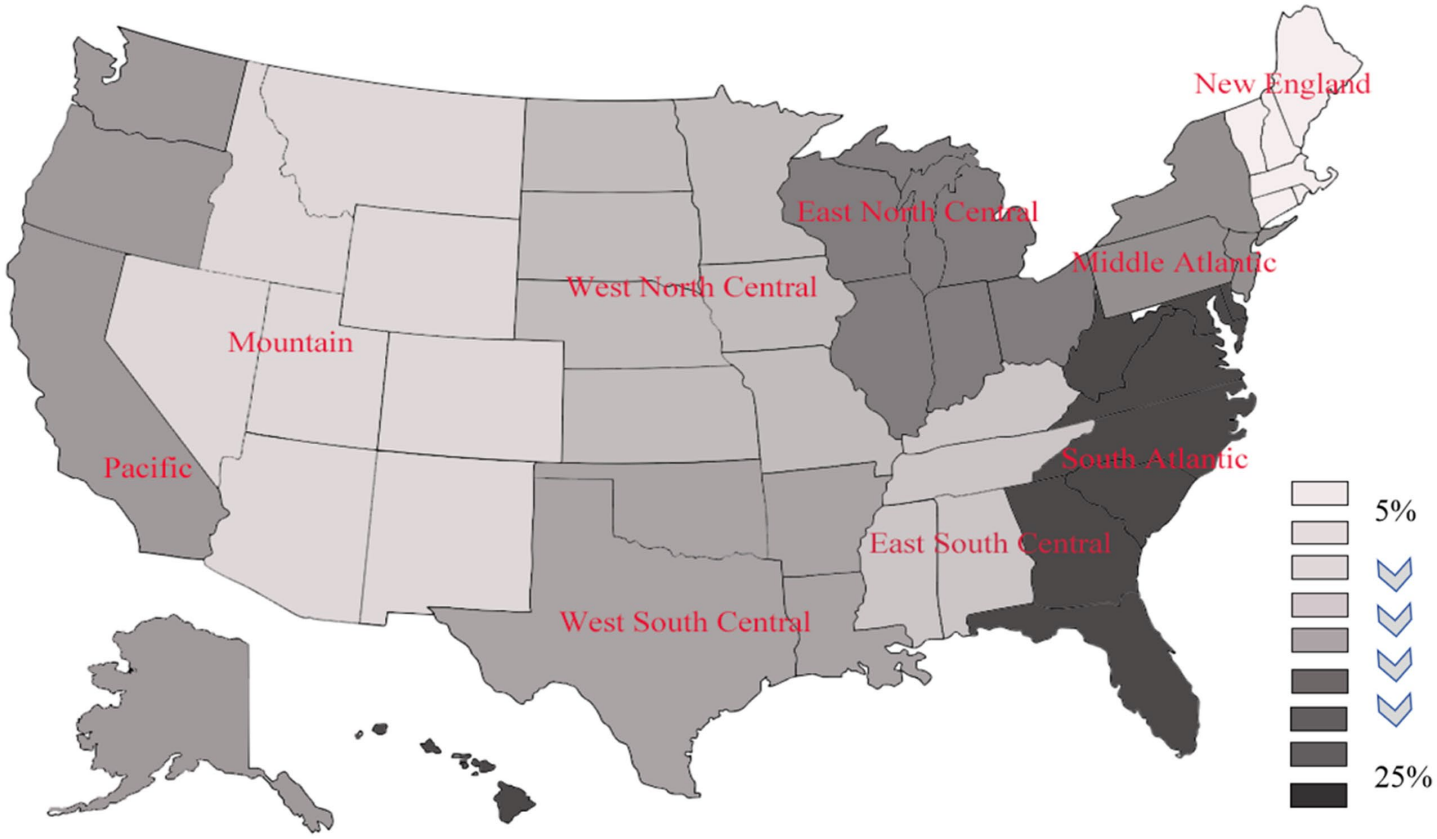
Regional distribution of nutritional anemia in the United States in 2020.

As shown in Figure 2, a notable concentration of nutritional anemia patients were located in the eastern region (>20%), especially with the highest distribution in the Atlantic region, followed by the northeastern region, and the lowest prevalence in the Mountain region as well as the northwestern region (Detailed values are given in Table 1).

### 3.4 Results of regression analysis of variables

The regression analysis outcomes were presented in Table 2, revealing a notable association (*p* < 0.001) between nutritional anemia and age, race, whether admission was chosen, location of the hospital, the presence of multiple injuries, length of hospitalization, mode of payment, income, and the transfer of the patient into and out of the hospital. While gender and month of admission yielded statistically insignificant findings. Regarding age and race, advanced age emerged as a risk factor, indicating that older individuals are more likely to develop nutritional anemia. Additionally, there is a higher prevalence of nutritional anemia among racial groups other than white race. Furthermore, a negative correlation was observed between factors such as economic income, length of hospitalization, and the prevalence of the disease.

**Table 2:** Association of adverse hospital outcomes in nutritional anemia.

Variables	Odds ratio	95% Confidence interval		*p*
		Lower	Upper	
AGE	1.002	1.001	1.003	<0.001
FEMALE	1.004	0.963	1.047	0.85
RACE	1.078	1.060	1.097	<0.001
AMONTH	1.004	0.998	1.009	0.19
ELECTIVE	0.189	0.171	0.210	<0.001
HOSP_DIVISION	0.975	0.967	0.984	<0.001
MULTINJURY	0.267	0.224	0.319	<0.001
LOS	0.939	0.932	0.947	<0.001
PAY	0.970	0.951	0.990	0.003
PL_NCHS	0.944	0.930	0.957	<0.001
TRAN_IN	0.902	0.858	0.947	<0.001
TRAN_OUT	0.880	0.852	0.908	<0.001
ZIPINC_QRTL	0.901	0.884	0.918	<0.001

HOSP_DIVISION, Location of the hospital; MULTINJURY, Multiple damage; LOS, Length of hospitalization; PAY, Payment Methods; PL_NCHS, Urban–rural divide; TRAN_IN, Transferred to hospital; TRAN_OUT, Transferred to other health units.

### 3.5 Statistics on other secondary diagnoses of patients with nutritional anemia

**Figure 3:**
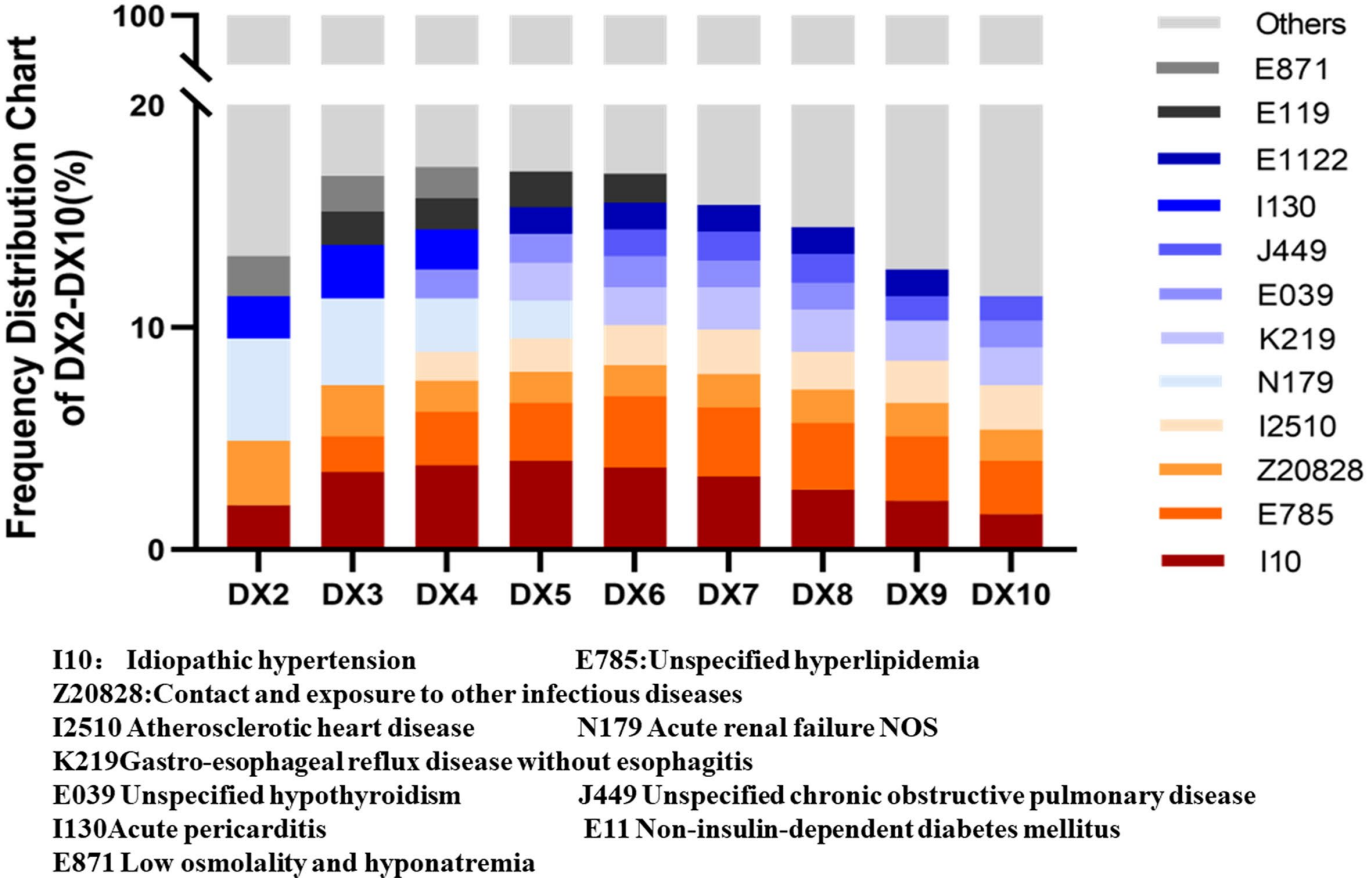
Distribution of the top 12 diseases.

Statistical findings of secondary diagnoses of 13,150 patients hospitalized with nutritional anemia revealed a substantial occurrence of underlying conditions, including hypertension, hyperlipidemia, and renal failure. The top 12 diseases in secondary diagnoses are presented in Figure 3, with specific details shown in Table 3. Notably, among top related 12 diseases, 26.9% of patients with nutritional anemia suffered from hypertension, 21.1% suffered from hyperlipidemia diseases, and 15.3% suffered from diseases such as infectious diseases.

**Table 3:** Frequency statistics of the top 12 diseases.

Disease code	ICD-10-CM Diagnosis:Other diagnoses 2 to 10										
	DX2	DX3	DX4	DX5	DX6	DX7	DX8	DX9	DX10	Totals	Valid percent
I10	258	459	506	523	493	440	354	291	208	3,532	26.9
E785	204	311	341	415	411	394	385	316	–*	2,777	21.1
Z20828	381	308	189	179	182	193	194	194	188	2008	15.3
I2510	267	237	266	249	250	202	166	225	–	1862	14.2
N179	605	508	318	224	–	–	–	–	–	1,655	12.6
K219	217	221	228	254	241	–	–	–	–	1,161	8.8
E039	185	152	177	172	158	153	–	–	–	997	7.6
J449	170	171	151	151	158	–	–	–	–	801	6.1
I130	312	234	248	–	–	–	–	–	–	794	6.0
E1122	157	153	157	160	163	–	–	–	–	790	6.0
E119	189	204	171	194	–	–	–	–	–	758	5.8
E871	207	180	239	–	–	–	–	–	–	626	4.8

–* denote no value; DXn, Other diagnostics; I10, Idiopathic hypertension; E785, Unspecified hyperlipidemia; Z20828, Contact and exposure to other infectious diseases; I2510, Atherosclerotic heart disease; N179, Acute renal failure NOS; K219, Gastro-esophageal reflux disease without esophagitis; E039, Unspecified hypothyroidism; J449, Unspecified chronic obstructive pulmonary disease; I130, Acute pericarditis; E1122, Non-insulin-dependent diabetes mellitus; E871, Low osmolality and hyponatremia.

### 3.6 Statistical results of secondary diagnoses of patients who died during hospitalization

**Figure 4:**
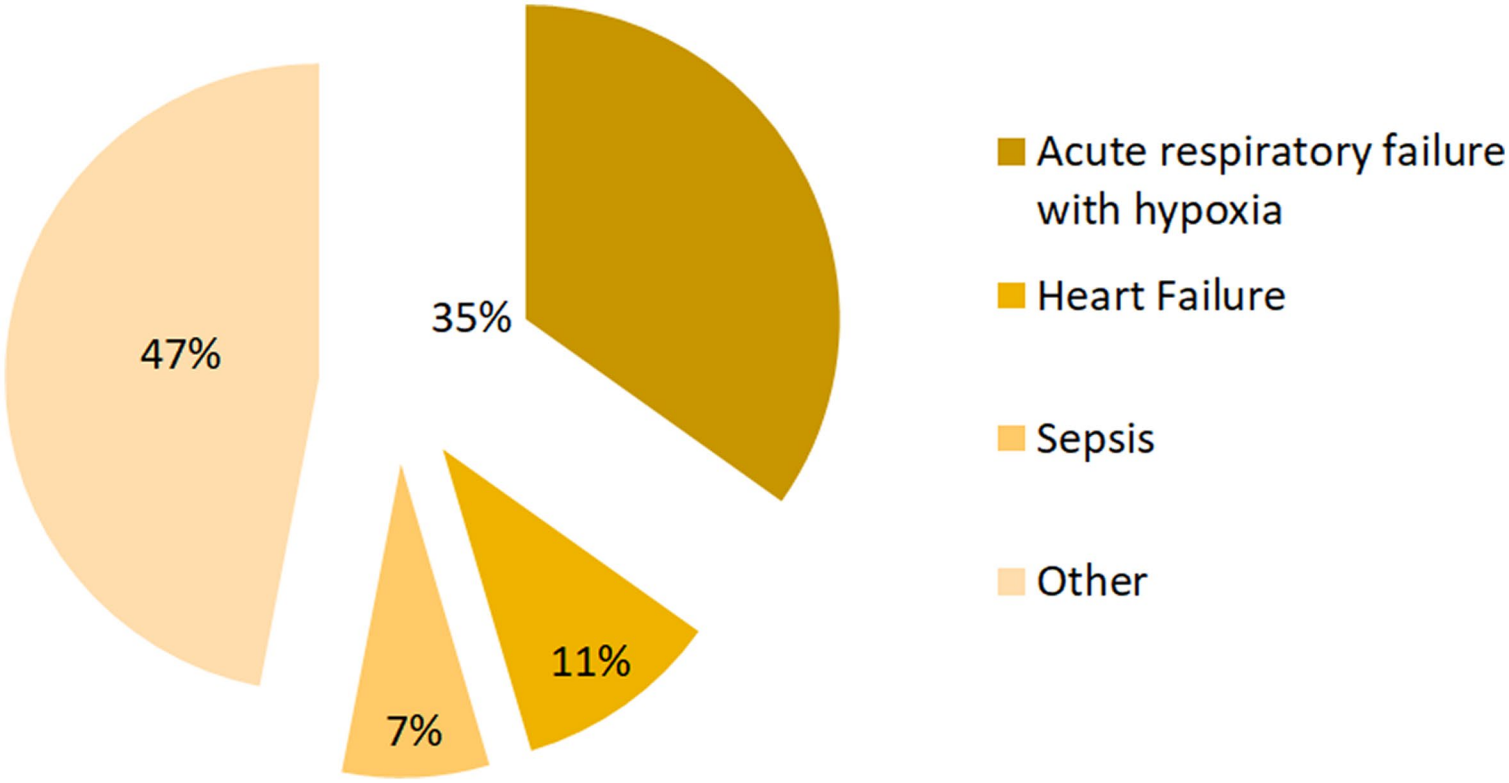
Secondary diagnosis in cases of death during hospitalization with nutritional anemia.

Among the 66 deaths from nutritional anemia (Figure 4), it was shown by their second diagnosis that 23 cases (34.85%) suffered from acute respiratory failure with hypoxia, 7 cases (10.61%) suffered from heart failure, and 5 cases (7.58%) suffered from sepsis (Figure 4).

## 4 Discussion

This article provides a comprehensive description of the inpatient burden among individuals with nutritional anemia based on cross-sectional analysis of a nationally representative hospitalization database. Our analysis of patient and hospital observations revealed that over one-fourth of hospitalized patients suffer from anemia, and nearly 5% of them have nutritional anemia. Specifically, more than 90% of patients hospitalized with nutritional anemia exhibited the iron-deficiency anemia subtype, with primarily affecting individuals over 65 years old. Hospitalizations were more frequent in females and in economically developed regions, particularly among low-income populations. Secondary diagnoses indicated a significant presence of cardiovascular and renal conditions, with acute respiratory and heart failures associated with fatal cases. Our findings warrant significant attention. We have identified that the prevalence of anemia among hospitalized patients remains unacceptably high. This calls for serious consideration within the healthcare sector. It is imperative to implement measures for the prevention and management of this issue. Additionally, gaining insights into the regional variations in anemia prevalence can facilitate more effective resource allocation and inform the development of healthcare policies.

Compared with previous studies (13, 15, 17), our research contributed significant insights into hospitalizations related to nutritional anemia. We conducted basic analyses to delve into the disease’s etiology and explored the impact of factors like age, gender, race, and economic conditions. The results showed that the number of nutritional anemia patients demonstrated a positive correlation with age, partially explaining the increase in hospitalizations and extended lengths of stay among older individuals. Additionally, age-related declines in bodily functions and immunity, along with reduced mobility, may account for the higher hospitalization rates among the older adult compared to young adults (18–20). Furthermore, the higher number of female patients can be attributed to physiological factors like menstrual loss and abnormal uterine bleeding (21, 22).

Economic conditions play a significant role in influencing nutritional anemia, where lower income is associated with higher prevalence. A notable difference in the prevalence of first quartile income was observed compared to the control group, consistent with existing literature (23, 24). This phenomenon could potentially be attributed to the limited access to healthy food in areas where economically disadvantaged and vulnerable populations reside (25, 26). In addition, the disparity in payment methods was evident through a lower prevalence of private insurance and a higher rate of Medicaid insurance compared to the control group, showcasing the potential impact of economic conditions on payment methods. This underscores the substantial influence of income on hospital admissions for nutritional anemia (27). Urbanization has been linked to an increase in anemia-related hospitalizations, with a notable concentration of nutritional anemia cases in the eastern and northwestern regions of the country. The urban low-income population constitutes the main population of hospitalization, which might be connected to life and work pressure, patients’ awareness of medical care, and the potential for reduced food expenditures due to financial stress among low-income urban individuals, ultimately leading to nutritional deficiencies. Moreover, certain remote and economically disadvantaged regions may see limited access to medical care due to financial constraints. This is all worth exploring in depth in future studies. Besides, hospitalizations for nutritional anemia in the United States are a gradual decline from 2016 to 2020, which is slightly different from the previous studies (13, 17). This decline could be attributed to the implementation of health policies and healthcare reforms in the previous years, alongside ongoing advancements in medical care and improving economic conditions. The main type of nutritional anemia is iron-deficiency anemia, which accounts for 92.88% of the prevalence. Addressing this, increasing the consumption of iron-rich foods or iron supplements stands out as a viable approach to mitigate anemia (28, 29).

In this study, we also found that nutritional anemia often coexisted with many other diseases, such as idiopathic hypertension, unspecified hyperlipidemia, infectious diseases, etc. Therefore, it is suggested individuals with nutritional anemia should pay close attention to monitoring the occurrence of these diseases. In addition, the second diagnosis of 66 deaths were 23 cases (34.85%) with acute respiratory failure with hypoxia and 7 cases (10.61%) with heart failure, which were the main comorbidities this may be associated with nutritional anemia (22, 30–34). Early detection of the causes of nutritional anemia and correction of malnutrition, nutritional supplementation and a balanced diet are key to preventing adverse outcomes. Many experts have indicated that investment in education can reduce the risk of anemia later in life, so raising awareness of nutritional health for all is a viable recommendation for reducing anemia (35–37). Currently, Nutritional counselling during antenatal care (38, 39), provision of micronutrients, management of family planning (40), dissemination of nutritional information and rational distribution of medical care for management of underlying chronic diseases are all feasible ways to reduce the prevalence of anemia (41).

Several limitations of this study should be acknowledged. Firstly, the focus of the article is limited to nutritional anemia, while other causes of anemia are not described. Secondly, the accuracy of the diagnostic results could not be independently verified. Thirdly, the presence of duplicate inpatients was not ascertainable, raising uncertainty regarding the exclusion of duplicated cases. Lastly, the severity of nutritional anemia in hospitalized patients were not available, which may result in overlooking the influence of severity when investigating the relationships between anemia and other variables.

## 5 Conclusion

In this study, we performed a descriptive analysis of nutritional anemia using the HCUP-NIS database. The findings revealed a notably high prevalence of anemia and nutritional anemia among hospitalized patients in the United States. Vulnerable groups, including older individuals and those with lower income levels, appeared to be at heightened risk for nutritional anemia. Furthermore, nutritional anemia frequently co-occured with various comorbidities such as hypertension, hyperlipidemia, etc. Hence, there is a pressing need to bolster public health management initiatives and enact relevant measures aimed at improving the overall health status of the population.

## Data availability statement

The original contributions presented in the study are included in the article/Supplementary material, further inquiries can be directed to the corresponding authors. The HCUP-NIS database is available for public access. (https://www.hcup-us.ahrq.gov/).

## Author contributions

JT: Formal analysis, Writing – original draft, Data curation. YF: Writing – original draft, Validation. XW: Investigation, Writing – original draft. JL: Conceptualization, Writing – original draft, Investigation. ZY: Data curation, Writing – original draft. XN: Supervision, Writing – review & editing. YZ: Funding acquisition, Project administration, Writing – review & editing.
