# Supplementary material for: Hospitalization of patients with nutritional anemia in the United States in 2020
Source: Front Public Health. 2024 May 13;12:1333069. doi: 10.3389/fpubh.2024.1333069 (PMC11128583; doi:10.3389/fpubh.2024.1333069)
Supplement: Supplementary file 1 [file Table_1.docx]

| **Nutritional anemia** | |
| --- | --- |
| **ICD-10-CM Code** | **ICD-10-CM Code Description** |
| **Iron deficiency** | |
| **D500** | Iron deficiency anemia secondary to blood loss (chronic) |
| **D501** | Sideropenic dysphagia |
| **D508** | Other iron deficiency anemias |
| **D509** | Iron deficiency anemia, unspecified |
| **Vitamin B12 deficiency** | |
| **D510** | Vitamin B12 defic anemia due to intrinsic factor deficiency |
| **D511** | Vit B12 defic anemia d/t slctv vit B12 malabsorp w protein |
| **D512** | Transcobalamin II deficiency |
| **D513** | Other dietary vitamin B12 deficiency anemia |
| **D518** | Other vitamin B12 deficiency anemias |
| **D519** | Vitamin B12 deficiency anemia, unspecified |
| **Folate deficiency** | |
| **D520** | Dietary folate deficiency anemia |
| **D528** | Other folate deficiency anemias |
| **D529** | Folate deficiency anemia, unspecified |
| **Other** | |
| **D530** | Protein deficiency anemia |
| **D531** | Other megaloblastic anemias, not elsewhere classified |
| **D532** | Scorbutic anemia |
| **D538** | Other specified nutritional anemias |
| **D539** | Nutritional anemia, unspecified |
